# Full-Thickness Macular Hole After Intravitreal Conbercept Injection in Branch Retinal Vein Occlusion

**DOI:** 10.7759/cureus.34660

**Published:** 2023-02-05

**Authors:** Pinxue Xie, Siquan Zhu, Ran Yan, Ping Wei, Xinxiao Gao

**Affiliations:** 1 Ophthalmology, Beijing Anzhen Hospital, Capital Medical University, Beijing, CHN; 2 Ophthalmology, Beijing Institute of Heart, Lung and Blood Vessel Diseases, Beijing, CHN; 3 Ophthalmology, Casey Eye Institute, Oregon Health & Science University, Portland, USA

**Keywords:** anti-vegf, intravitreal injections, conbercept, macular hole, branch retinal vein occlusion

## Abstract

This article reports a case of macular hole (MH) formation following intravitreal conbercept injection for branch retinal vein occlusion (BRVO). A 70-year-old male received three consecutive intravitreal injections of conbercept for the treatment of macular edema secondary to BRVO in his left eye. Due to the outbreak of the COVID-19 epidemic, the patient was lost to follow-up. At two months follow-up, a full-thickness MH was detected by fundoscopic and optical coherence tomography examination. Fortunately, the MH was successfully closed after pars plana vitrectomy. MH is a rare complication following intravitreal injections for RVO, which should be considered by clinicians.

## Introduction

Macular edema is a common complication of retinal vein occlusion (RVO), which can cause severe visual impairment [1,2]. At present, anti-VEGF (aVEGF) injection is the first-line treatment with favorable results, however, some adverse effects have been reported in a few cases, including macular hole (MH) [3-5]. This retrospective case report presents a full-thickness MH following intravitreal aVEGF injection.

## Case presentation

**Figure 1:**
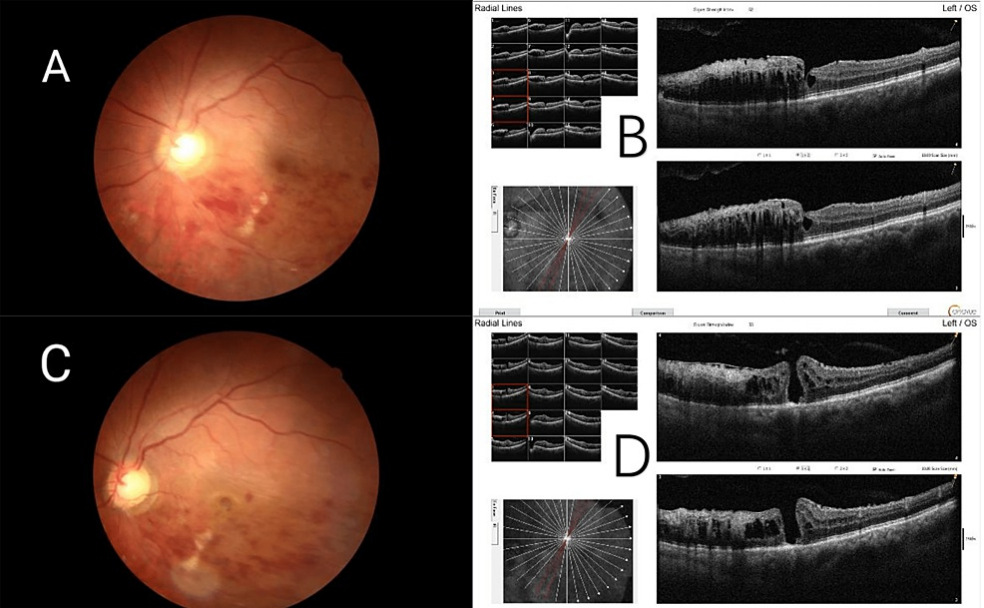
Pre-surgery fundus color images and OCT images OCT: Optical coherence tomography A: Color fundus photograph showing inferior-temporal branch retinal vein occlusion in the left eye. B: Spectral-domain optical coherence tomography (SD-OCT) revealed cystoid changes with thickened retina. C: Color fundus photograph showing branch retinal vein occlusion with the formation of a macular hole. D: OCT examination demonstrated a full-thickness macular hole accompanied by the epiretinal membrane and intraretinal cysts.

A 70-year-old male was referred to our clinic with a complaint of blurred vision in both eyes for more than three months. The best corrected visual acuity (BCVA) was 20/40 in the right eye and 20/100 in the left eye with normal intraocular pressure. Anterior segment examination was unremarkable except for lens opacity in both eyes. A biomicroscopic examination of the fundus revealed inferior-temporal branch retinal vein occlusion (BRVO) with retinal hemorrhage in the left eye. Spectral-domain optical coherence tomography (SD-OCT) revealed cystoid changes with thickened retina. The patient had a history of hypertension for 10 years, but no diabetes mellitus or hyperlipidemia. Phacoemulsification combined with intraocular lens implantation was performed on both eyes due to the cataract. Then the left eye was treated with an intravitreal injection of conbercept (0.5 mg) every month for three consecutive months. Due to the recurrence of macular edema, scattered laser photocoagulation was performed on the inferior temporal retina in the left eye. At the follow-up one month later, the BCVA was 20/80 in the left eye. Optical coherence tomography (OCT) examination revealed recurrent cystoid macular edema, with a thin inner wall of the foveal cystoid space (Figures 1A-1B). Another injection of aVEGF was planned. Unfortunately, the outbreak of the COVID-19 epidemic took place soon. During that period, the patient was lost to follow-up. Two months later, he came back with the complaint of decreased visual acuity and central scotoma in the left eye. BCVA was reduced to 20/400. Fundoscopic and OCT examinations confirmed a full-thickness MH accompanied by the epiretinal membrane (ERM) and intraretinal cysts in the left eye (Figures 1C-1D).

**Figure 2:**
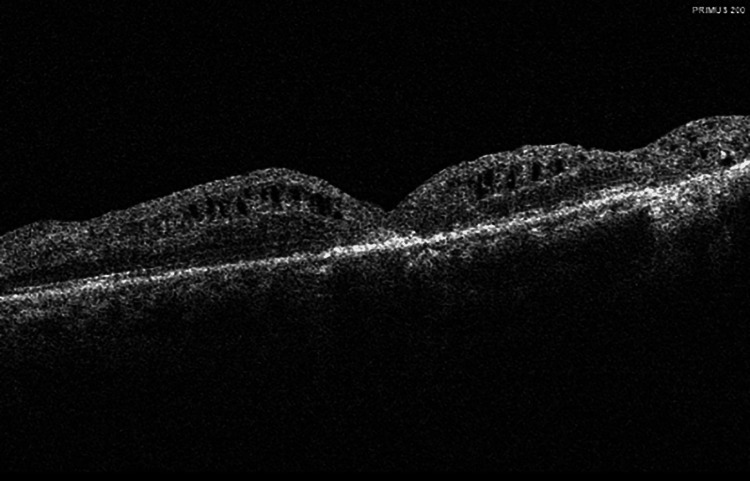
OCT showed anatomical recovery after treatment. OCT: Optical coherence tomography Six months following pars plana vitrectomy, OCT revealed successful closure of the macular hole and decreased central macular thickness in the left eye.

After obtaining informed consent from the patient, a 23 gauge pars plana vitrectomy (PPV) was performed, combined with internal limiting membrane peeling and gas tamponade. At the time of vitrectomy, posterior vitreous detachment (PVD) was noticed following staining with triamcinolone acetonide. Six months after surgery, OCT showed the successful closure of MH (Figure 2). Central retinal thickness (CRT) decreased to 227 um and BCVA improved to 20/80.

## Discussion

We report a patient who developed a full-thickness MH after intravitreal aVEGF injections for the treatment of BRVO. Our literature review revealed only two cases presenting a similar occurrence, who received ranibizumab and triamcinolone acetonide injections respectively [3,4]. Another publication describes the progression of the macular hole following the injection of bevacizumab for hemicentral RVO [5]. To the best of our knowledge, this is the first report of MH formation subsequent to intravitreal conbercept injection for BRVO.

Several possible mechanisms might be hypothesized to explain MH formation in our patient. Retinal ischemia and cystoid degeneration due to the circulatory disturbance, may induce the thinning of the inner wall at the foveal cystoid space and subsequently make it more susceptible to MH formation. Another possible explanation is that secondary ERM may occur after retinal photocoagulation, and aVEGF therapy can further stimulate the formation of fibrosis. Contraction of the ERM may lead to the exacerbation of tangential traction on the damaged macular retina, which might have been a possible cause of MH [6]. Alternatively, as in prior reports, the possibility of antero-posterior traction exerted by vitreous incarceration at the injection site combined with rapid shrinkage of macular thickness cannot be completely ruled out. However, in our case, vitreous macular traction might not have been a causative factor for MH formation, since complete PVD was observed in the OCT. Moreover, PVD was further confirmed during the surgery. Interestingly, a recent study has suggested that elderly age, cystoid macular edema and anomalous vitreoretinal interface may be associated indicators for the formation of full-thickness MH in eyes treated with aVEGF for CRVO [7]. Similarly, all these potential risk factors can be found in our case. It has been reported that MH secondary to RVO may achieve satisfactory results after surgery [2,3]. In agreement with these studies, our case also showed favorable morphological outcomes and improved visual function following PPV surgery.

## Conclusions

To sum up, a macular hole is a rare complication following intravitreal aVEGF injection for RVO. Our patient achieved good anatomical recovery after treatment of MH complications. Early recognition of this infrequent adverse effect and intervention with vitrectomy may benefit to the final visual prognosis.
